# Route of Glucose Uptake in the Group a Streptococcus Impacts SLS-Mediated Hemolysis and Survival in Human Blood

**DOI:** 10.3389/fcimb.2018.00071

**Published:** 2018-03-14

**Authors:** Ganesh S. Sundar, Emrul Islam, Rezia D. Braza, Aliyah B. Silver, Yoann Le Breton, Kevin S. McIver

**Affiliations:** Department of Cell Biology and Molecular Genetics, Maryland Pathogen Research Institute, University of Maryland, College Park, College Park, MD, United States

**Keywords:** group A streptococcus, glucose uptake and metabolism, Streptolysin S, pathophysiology, phosphotransferase system

## Abstract

The transport and metabolism of glucose has been shown to have far reaching consequences in the transcriptional profile of many bacteria. As glucose is most often the preferred carbon source for bacteria, its presence in the environment leads to the repression of many alternate carbohydrate pathways, a condition known as carbon catabolite repression (CCR). Additionally, the expression of many virulence factors is also dependent on the presence of glucose. Despite its importance, little is known about the transport routes of glucose in the human pathogen *Streptococcus pyogenes*. Considering that *Streptococcus pyogenes* is an important human pathogen responsible for over 500,000 deaths every year, we characterized the routes of glucose transport in an effort to understand its importance in GAS pathogenesis. Using a deletion of glucokinase (Δ*nagC*) to block utilization of glucose imported by non-PTS pathways, we determined that of the two glucose transport pathways in GAS (PTS and non-PTS), the non-PTS pathway played a more significant role in glucose transport. However, the expression of both pathways is linked by a currently unknown mechanism, as blocking the non-PTS uptake of glucose reduces *ptsI* (EI) expression. Similar to the effects of the deletion of the PTS pathway, lack of the non-PTS pathway also leads to the early activity of Streptolysin S. However, this early activity did not adversely or favorably affect survival of Δ*nagC* in whole human blood. In a subcutaneous murine infection model, Δ*nagC*-infected mice showed increased lesion severity at the local site of infection; although, lesion size and dissemination from the site of infection was similar to wild type. Here, we show that glucose transport in GAS is primarily via a non-PTS pathway. The route of glucose transport differentially affects the survival of GAS in whole human blood, as well as the lesion size at the local site of infection in a murine skin infection model.

## Introduction

Glucose is abundantly found in nature and represents a rapidly metabolized carbon and energy source for most living organisms, including bacteria. Carbon catabolite repression (CCR) represents an important global control system in bacteria and other microorganisms that allows for preferred utilization of glucose and other preferred energy sources by inhibiting the uptake and metabolism of less efficient alternative carbon sources. In the human body, glucose is present in 32 of 43 biofluids or tissues studied so far, with the highest glucose concentrations usually found in blood (Wishart et al., 2007, 2009, 2013). As such, bacterial pathogens that infect the human body possess many mechanisms to transport and metabolize glucose (Cvitkovitch et al., 1995; Vitko et al., 2016). For example, glucose transport in the Gram-positive pathogen *Staphylococcus aureus* has been shown to be highly redundant, involving four different transporter proteins (Vitko et al., 2016). Even a quadruple *S. aureus* mutant lacking these uptake systems had residual glucose metabolism, indicating the potential for an additional unidentified transporter (Vitko et al., 2016). Thus, utilization of glucose during infection of the human host is extremely important for successful colonization by bacterial pathogens.

*Streptococcus pyogenes* (Group A Streptococcus, GAS) is a strict human pathogen that normally colonizes the nasopharyngeal mucosa or the skin (Cunningham, 2000). These infections are superficial, generally self-limited, and are treated effectively with antibiotics. However, these infections can lead to the development of post-infection autoimmune sequelae. Problems arise when these infections gain access to sterile sites via the bloodstream, leading to poor clinical outcomes. Such invasive infections are difficult to treat, and often lead to life-threatening symptoms such as necrotizing fasciitis, streptococcal toxic shock syndrome, acute rheumatic fever, and glomerulonephritis (Cunningham, 2000). Neurological disorders have also been shown to occur, such as Tourette's, tics, and pediatric autoimmune neuropsychiatric disorders associated with streptococcal infections (PANDAS) (Esposito et al., 2014). Altogether, invasive GAS infections and sequelae kill nearly half a million people worldwide every year (Carapetis et al., 2005). Without a licensed vaccine, extreme importance has been given to understanding the host-pathogen dynamics of *Streptococcus pyogenes*.

In general, GAS relies heavily on glycolysis for the fermentation of incoming sugars, as it contains an incomplete TCA cycle and does not carry out cellular respiration. The pathway is made up of almost entirely essential proteins, highlighting its importance (Le Breton et al., 2015). Since GAS is a lactic acid bacterium, pyruvate is generally converted to lactate/lactic acid by L-lactate dehydrogenase (Ldh), causing acidification of the surrounding media. GAS possess several sugar metabolic systems that allow for the transport and fermentation of sugars, including ABC transporters, the PTS, and parts of the Entner-Doudoroff and Pentose Phosphate pathway.

Many sugar metabolic pathways have been linked to the virulence of pathogens (Foster et al., 2003; Petzold et al., 2006; Shelburne et al., 2008b; Lowe et al., 2009; Ngamskulrungroj et al., 2009; Tournu et al., 2013; López-Garrido et al., 2015). One such system, the phosphoenolpyruvate-dependent phosphotranferase system (PTS), is a phosphorelay system that incorporates general and sugar-specific enzymes to effectively couple the translocation and phosphorylation of an incoming sugar. Enzyme I (EI) and HPr are the two general proteins common to the use of all PTS carbohydrates, where EI is phosphorylated as PEP is converted to pyruvate, which will then go on to phosphorylate HPr on its Histidine-15 residue. Although the entire PTS is found in most bacteria, the number of sugar-specific enzymes varies. Sugar-specificity is brought about through Enzyme IIs (EIIs), which are made up of 3–4 subunits. Each EII generally contains an EIIA domain, which is phosphorylated by HPr, and an EIIB domain, which is phosphorylated by EIIA, and an EIIC/D domain, which transports the sugar into the cell, and are phosphorylated by EIIB. Not all Enzyme IIs have an EIID domain, as only mannose-family EIIs have been shown to possess them. The PTS also participates in signal transduction through the actions of EIIs and HPr. In the absence of the inducing sugar, phosphates that reside on EIIBs can be transferred to PTS-regulatory domains found on several transcriptional regulators, thereby changing their activity. HPr kinase (HprK) will phosphorylate Hpr on its Serine-46 residue in the presence of the preferred carbon source, usually glucose, which in conjunction with the carbon catabolite protein A (CcpA) will elicit carbon catabolite repression. These two mechanisms allow the PTS to influence the expression of a wide variety of genes in response to carbohydrates available in the environment. In contrast, non-PTS uptake pathways (e.g., permeases) import glucose directly and require phosphorylation by a cytoplasmic glucokinase in order to enter glycolysis.

Streptolysin S (SLS), a GAS toxin that lyses host tissues and blood cells, was shown to be under the influence of the PTS, as a PTS-null (Δ*ptsI*) mutation led to earlier SLS activity as compared to the parental strain MGAS5005 (Gera et al., 2014). We screened an annotated EIIC mutant library in a previous study, and discovered 7 out of 14 EIIC mutants that had earlier hemolysis as compared to MGAS5005 (Sundar et al., 2017). We carried out this screen in an effort to determine if the lack of utilization of a particular PTS carbon source was responsible for the dysregulation of timely SLS-mediated hemolysis. There was no carbohydrate whose metabolism was commonly effected in all 7 EIIC mutants which displayed early hemolysis (Sundar et al., 2017). However, all of these EIIs do appear in the CcpA regulon (DebRoy et al., 2016). Considering that MGAS5005.Δ*ccpA* also exhibits early hemolysis (Kinkel and McIver, 2008), we hypothesized that disruption of efficient glucose metabolism could be the trigger for SLS-mediated hemolysis.

In GAS, glucose is hypothesized to be transported by the PTS. Several EIIs in other bacteria have been shown to transport glucose, and deletion of certain PTS transporters in GAS leads to altered metabolism of glucose (Castro et al., 2009; Vitko et al., 2016; Sundar et al., 2017). However, the specific EIIs responsible for glucose transport in GAS are unknown, as the only annotated glucose-specific EII gene *malT* (formerly *ptsG*) has been shown to transport maltose instead (Shelburne et al., 2008a). Glucose can also be transported by non-PTS pathways. In *Staphylococcus xylosus*, a glucose uptake protein (GlcU) was identified as a non-PTS glucose transporter, which has 32% identity to *glcU* in *Streptococcus pyogenes* (Fiegler et al., 1999). In *Lactococcus lactis*, GlcU was shown to rely on proton-motive force to transport glucose into the cell, and was considered a low-affinity transporter (Castro et al., 2009). These pathways would require the imported unphosphorylated glucose to be phosphorylated by other kinases at the expense of ATP, usually a glucokinase (GlkA). The GAS genome encodes for a glucokinase (*nagC*), and *glcU*. Metabolism of glucose through the PTS and the glucokinase are hypothesized to be the only two routes of glucose metabolism, as in *S. aureus*, a Δ*glk*Δ*ptsH-H15A* mutant [a strain that cannot transport glucose through the PTS or be phosphorylated by the glucokinase (GlK)], does not grow on glucose (Vitko et al., 2016). In this study, we characterized the routes of glucose metabolism and compared their influence in SLS-mediated hemolysis, GAS survival in whole human blood, and GAS pathogenesis in a murine skin infection model.

## Materials and methods

### Bacterial strains and media

*Streptococcus pyogenes* (GAS) serotype M1T1 strains MGAS5005 (Sumby et al., 2005) and 5448 (Chatellier et al., 2000) were isolated from patients with invasive GAS infections. Strains were grown in Todd-Hewitt medium supplemented with 0.2% yeast extract (THY) or in chemically defined media (CDM; Alpha Biosciences). Sodium bicarbonate (59.51 μM) and L-cysteine (11.68 μM) was added fresh to CDM before use. The carbon source (0.5% glucose or 1% other PTS sugars) was also added prior to GAS inoculation. *Escherichia coli* strain DH5α (*hsdR17 recA1 gyrA endA1 relA1*) was used as a recipient for constructing mutagenic plasmids and grown in Luria-Bertani (LB) broth. Spectinomycin (Sp) was added to growth medium at a concentration of 100 μg/ml for both GAS and *E. coli*. Kanamycin (Km) was added at 50 μg/ml for *E. coli* and 300 μg/ml for GAS.

### DNA manipulations

PCR was performed using Accuprime Pfx (Life Technologies) for cloning and Taq DNA polymerase (NEB) for diagnostic assays, according to their manufacturer's protocols. DNA sequencing was carried out by Genewiz, Inc. Plasmids were isolated from *E. coli* using the Wizard Plus SV miniprep kit (Promega). Genomic DNA was extracted from GAS using the Master-Pure complete DNA and RNA purification kit for Gram-positive bacteria (Epicenter). DNA fragments in agarose gels were purified using the Wizard SV gel and PCR cleanup kit (Promega).

### Generation of glucose metabolic pathway mutants

A *glcU* polar insertional inactivation mutant (MGAS5005.Δ*glcU*, Supplementary Table 1) was created using the pCRS system (Supplementary Table 2) (Le Breton et al., 2013) since it is monocistronic. A non-polar *nagC* mutant (Δ*nagC*, Supplementary Table 1) was generated by replacing *nagC* with a kanamycin resistant cassette (*aphA3*) using allelic exchange as previously described (Le Breton and McIver, 2013). Revertants or rescues were saved that were sensitive to both drugs, indicating reversion of the merodiploid mutation to a wild type *nagC* allele. A reduced amount of kanamycin (50 μg/μl) was used for passaging due to weak expression from the *nagC* promoter. A double mutant MGAS5005.Δ*glcU*Δ*ptsI* was generated by integration of a pCRK-derived plasmid (Supplementary Table 2) possessing a region of homology from *glcU* into the MGAS5005.Δ*ptsI* genome (Gera et al., 2014).

### Carbon growth assays

#### Growth analysis of GAS in CDM or C-media plus a PTS carbohydrate source

Growth of GAS was measured as described previously (Sundar et al., 2017). Briefly, GAS cells were grown overnight on blood agar plates, resuspended in saline, and adjusted to an OD_600_ of 0.2 before being inoculated in chemically defined media (CDM) or C-media plus the tested PTS carbohydrate. C-media was prepared as previously described (Lyon et al., 1998) 25 μL aliquots were then added to 500 μL of growth media (CDM or C-media with sugar added) to each well of a 48-well plate (Corning/Costar). Growth curves were also analyzed as described previously (Sundar et al., 2017). Briefly, OD_600_ of the mutants were compared to wild type (% WT) for each growth curve. The median and interquartile range was determined for each mutant data set in each PTS carbohydrate tested. ΔOD_600_ were calculated by taking the max OD_600_ at each time interval and subtracting the initial ΔOD_600_. Data shown is the average of at least three biological replicates.

#### Glucose consumption

Glucose consumption of GAS strains grown in CDM or THY were calculated using a blood glucometer as follows: GAS was grown overnight in THY, pelleted and washed in saline, and adjusted to an OD_600_ of 0.2 in saline. Next, cells were inoculated 1:20 in 5 ml THY or CDM + 0.5% glucose. Sugar concentrations were determined as mg/dl at 0 and 24 h using a blood glucometer (AimStrip ® Plus) according to the manufacturer's protocol. To calculate the total amount of glucose consumed, the drop in the concentration of glucose at the end of 24 h was assessed, yielding the total mg of sugar used during GAS growth in a particular media.

### Carbon metabolic profiles

Carbon metabolic profiles were determined using the API®50 CH system (Biomérieux) as described previously (Valdes et al., 2016; Sundar et al., 2017). Briefly, strains were cultured overnight on blood agar plates, suspended in saline, and vortexed for 3 min. Strains were then diluted and added to 10 ml of API 50 CHL medium. This was then added to 50 cupules present in the assay kit. Utilization scores were determined at 24 and 48 h, with utilization of a carbon source given a “+,” partial utilization given a “±,” and no utilization given a “−.” Utilization scores are the sum measure of both readings for each carbon source tested, as described in Sundar et al. (2017). Briefly, “+” are scored as 1, “±” are scored as 0.5, and “−” are scored as 0.

### qRT-PCR

Quantitative real-time PCR (qRT-PCR) experiments were performed on total RNA isolated from GAS strains grown in THY to late logarithmic phase (Klett ~ 100) as described previously using a Triton-X protocol (Sung et al., 2003). Isolated RNA was treated with DNAse I, then 25 ng was added to SYBR green master mix (Applied Biosystems) with 6.5 μl of each real-time primer (20 nM stock) using the one-step protocol on a Light Cycle 480 (Roche). Primer3 (http://biotools.umassmed.edu/bioapps/primer3_www.cgi) was used to design real-time primers (Supplementary Table 3). Data is shown as the average of at least three biological replicates, with at least 2-fold expression change representing significance. Data was analyzed using the E-method for relative quantification analysis on the Roche LC480.

### Hemolysis assay

Hemolytic activity was determined by measuring the amount of lysed red blood cells (RBCs) as previously described (Gera et al., 2014). Briefly, GAS was grown in THY + 10% heat-inactivated horse serum, with samples taken every hour for up to 8 h. Samples were then pelleted and 50 μl of the resulting culture supernatant was added to 950 μl of defibrinated sheep red blood cells (RBCs) prepared as previously described (Gera et al., 2014). RBCs with added culture supernatant were then incubated for 1 h at 37°C in 1.5 ml Eppendorf tubes. Tubes were then centrifuged at 3,000 × *g* for 10 min to clear intact RBCs. To measure the amount of RBC lysis (hemoglobin release), the OD_541_ was taken for each sample. Data shown is the compilation of at least three biological replicates.

### Lancefield bactericidal assay

All blood donation was approved by the University of Maryland Institutional Review Board (IRB) (protocol 10-0735) with written informed consent from donors and records archived. To monitor GAS survival in whole human blood, a Lancefield bactericidal assay was performed as previously described (Lancefield, 1957). Briefly, GAS was grown to early exponential phase (OD_600_ ~ 0.1) and serially diluted in saline. Fresh heparinized whole human blood was incubated with 50 μl of a 10^−4^ dilution of GAS suspension (*ca*. 50 to 200 CFUs). Whole human blood with GAS was then incubated at 37°C with rotation for 3 h. The multiplication factor (MF) was calculated as a ratio of the GAS CFU counts after and before blood challenge. Data is shown as a percent WT, where the MF from MGAS5005 was divided by the MF of the mutant strains. Data shown represents the average of at least 3 biological replicates. Significance was determined using an unpaired Student's *t*-test.

### Ethics statement

All animal work was carried out in accordance with the Institutional Animal Care and Use Committee (IACUC) at the University of Maryland, College Park (protocol R-16-05) in an Association of Assessment and Accreditation of Laboratory Animal Care (AAALAC)-accredited ABSL-2 facility. Humane treatment of animal subjects was done in accordance with the Office of Laboratory Animal Welfare (OLAW) at NIH, Public Health Service, and the Guide for the Care and Use of Laboratory Animals guidelines. Extreme care was taken to limit the pain and distress to the animals.

### Subcutaneous murine infection model

The murine skin infection model was carried out as described previously (Gera et al., 2014). Briefly, GAS cultures grown overnight were inoculated at a dilution of 1:20 in 75 ml of THY and incubated until late-logarithmic phase (Klett 100) statically at 37°C. 5 to 7-week-old female CD-1 mice (Charles River Laboratories) were infected with 3 × 10^9^ CFU/ml, as determined by microscope counts and later verified by viable counts on THY plates. Ketamine was used to anesthetize mice, hair was removed from a 3-cm^2^ area of one of the haunches with Nair (Carter Products). 100 μl of GAS suspension (saline) was injected subcutaneously. Each mouse was monitored three times daily for 7 days. Upon signs of morbidity, mice were euthanized by CO_2_ asphyxiation. Lesion sizes were measured 36 h post infection using Image J. Lesion severity was monitored as previously described (Sundar et al., 2017) by determining the mean red pixel intensity among the pixels present in a lesion selection. Significance was determined using an unpaired two-tailed *t*-test (*p*-value < 0.05). Survival significance was determined by Mantel-Cox log rank test (*p*-value < 0.05), using GraphPad Prism software.

## Results

### A GAS PTS mutant does not regulate hemolytic activity based on sugar concentration

**Figure 1:**
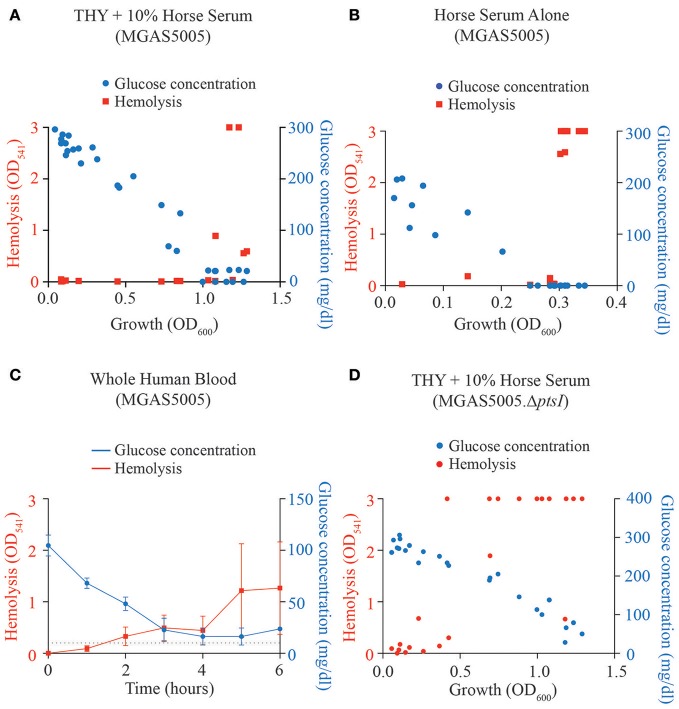
Hemolytic activity correlates to low glucose concentration. Glucose concentration (blue) and hemolysis (red) were determined over growth (OD_600_) as described in section Materials and Methods for MGAS5005 grown in **(A)** THY + 10% horse serum, **(B)** horse serum alone and **(C)** whole human blood. **(D)** Glucose concentration and hemolysis were assayed as above for the PTS mutant MGAS5005.Δ*ptsI* grown in THY + 10% horse serum.

Previous studies from our group showed that an M1T1 MGAS5005.Δ*ptsI* (ΔEI) mutant exhibited early onset of hemolytic activity during growth as compared to the parental MGAS5005 (Gera et al., 2014), likely due to the lack of access to a PTS carbohydrate. However, our recent mutant screen of GAS PTS EII components found 7 EII loci that exhibited hemolytic activity early in growth yet did not affect the metabolism of a particular carbohydrate (Sundar et al., 2017). Interestingly, all of these EII loci are part of the published CcpA regulon (DebRoy et al., 2016). Although MGAS5005 only exhibits hemolytic activity at late log-stationary phase when preferred sugar concentrations are low, a direct correlation between sugar concentration and hemolytic activity has not been explicitly shown in GAS. Therefore, we grew MGAS5005 in media or biofluids representing three glucose amounts and assayed at which glucose concentration that hemolysis begins to occur. Glucose concentrations and hemolytic values were measured over growth (OD_600_) for MGAS5005 grown in THY +10% horse serum (Figure 1A), horse serum alone (Figure 1B), and in whole human blood (Figure 1C). In all three conditions, parental MGAS5005 did not display SLS-mediated hemolysis until glucose concentrations were around 50 mg/dl or lower. In the Δ*ptsI* mutant, hemolysis is detectable at glucose concentrations higher than that of wildtype in all three medias (Figure 1D), suggesting that the lack of flux through glucose metabolism may be the trigger for SLS-mediated hemolysis.

### Glucose metabolism occurs primarily through the non-PTS glucokinase NagC

**Figure 2:**
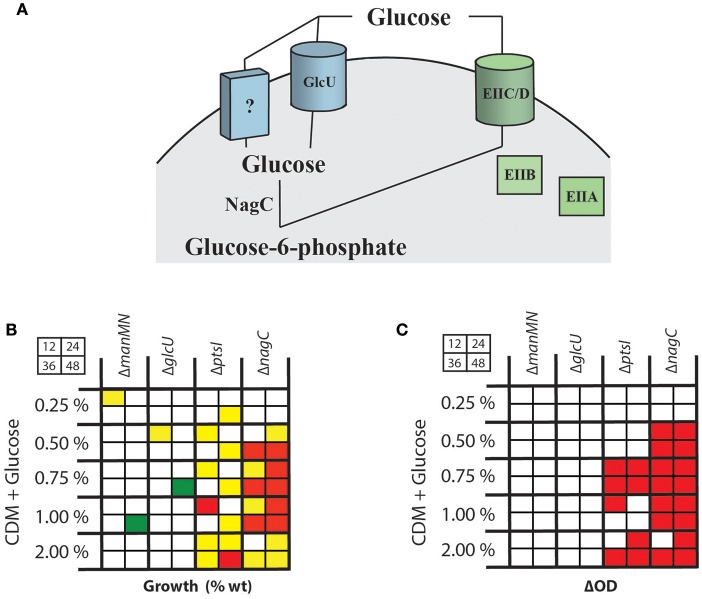
Characterization of pathways for glucose uptake in MGAS5005. **(A)** Schematic of predicted PTS (green) and non-PTS (blue) pathways for glucose transport in GAS leading to glucose-6-phosphate. Indicated are a putative glucose-specific PTS EII system, a predicted non-PTS GlcU glucose permease and the NagC glucokinase. **(B)** Growth assays (%WT) at 12, 24, 36, and 48 h (as shown in grid) were performed on GAS mutants in the entire PTS *(*Δ*ptsI*), a PTS EIICD locus (Δ*manMN*) important for glucose utilization in other streptococci, the GlcU permease (Δ*glcU*) and the NagC glucokinase *(*Δ*nagC*) were grown in CDM + various % glucose as described in section Materials and Methods. Growth above WT (green), 2-fold below WT (yellow), < 2-fold below WT (red), and similar to WT (white) are indicated. **(C)** ΔOD_600_ was calculated for the same mutants compared to MGAS5005 and outcomes indicated as described for **(B)**. Red boxes indicated total yields statistically significantly below wildtype (*p* < 0.05), determined using a student's *t*-test.

Since regulation of SLS-mediated hemolysis does not seem to be affected by the metabolism of a specific PTS carbohydrate, but is impacted by the disruption of glucose utilization, we wanted to characterize the routes of glucose uptake and metabolism in MGAS5005. Currently, little is known about glucose utilization in GAS. We previously found that Δ*ptsI* has no growth defect in CDM + 0.5% glucose (Gera et al., 2014), suggesting that the PTS system either does not transport glucose or that an alternate glucose transport system exists that is specific to glucose in conjunction with the PTS (Figure 2A). To test this hypothesis, we constructed mutants in a putative non-PTS glucose transporter (*glcU*, M5005_Spy1856) based on its role in other streptococcal species (Vitko et al., 2016), and a putative glucokinase (*nagC*, M5005_Spy1311) that would be required to generate glucose-6-phosphate following non-PTS glucose import (Figure 2A). For both mutants, rescued strains were created by restoring their wild type genotype.

WT MGAS5005, Δ*glcU*, Δ*nagC*, and Δ*ptsI* GAS strains were grown in THY, a glucose-rich media, to evaluate growth defects of each mutant. A Δ*manMN* (ΔMannose) mutant was included based on studies suggesting that a homologous mannose-specific EII is a high affinity glucose transporter in *S. pneumoniae* (Fleming and Camilli, 2016). Total yield (ΔOD_600_) was calculated after 24 h was similar for all strains tested (Supplementary Figure 1). However, the total amount of sugar consumed (see section Materials and Methods) was slightly lower in Δ*manMN* and Δ*glcU* as compared to MGAS5005 (Supplementary Figure 1). Since THY is a complex rich media that potentially contains multiple sugars, we tested the same strains in CDM + 0.5% glucose and monitored the amount of sugar consumed to determine if loss of any of these predicted glucose utilization pathways reduced the efficiency of glucose metabolism. None of the mutants showed significantly decreased glucose consumption when the total yield was standardized for all strains (Supplementary Figure 1).

We next grew each mutant in CDM with different concentrations of glucose as the sole carbon source for 48 h, calculating growth (%WT) and total yield (ΔOD_600_) at 12, 24, 36, and 48 h as described in section Materials and Methods. The Δ*manMN* and Δ*glcU* mutants had very little effect on GAS growth on glucose (Figure 2B, Supplementary Figures 2A,B). The Δ*ptsI* mutant exhibited a consistent growth defect when grown in 2% glucose (Figure 2B, Supplementary Figure 2C), while at 0.5, 0.75, and 1% glucose, growth was only affected early (12 h) and late (48 h) in growth (Figure 2B). However, when looking just at the total yield, growth of the Δ*ptsI* mutant on 0.75% glucose exhibited the most drastic reduction in growth (Figure 2C, Supplementary Figure 3). In contrast, Δ*nagC* had the lowest growth compared to WT of all of the mutants tested in all concentrations of glucose (Figures 2B,C, Supplementary Figures 2D, 3). This would suggest that either the main route of glucose transport occurs through a non-PTS glucose transport system, or that deleting the glucokinase gene (Δ*nagC*) somehow affects the expression of the PTS system, hindering overall glucose transport.

### Loss of *nagC* reduces the ability of GAS to grow on certain PTS carbohydrates

**Figure 3:**
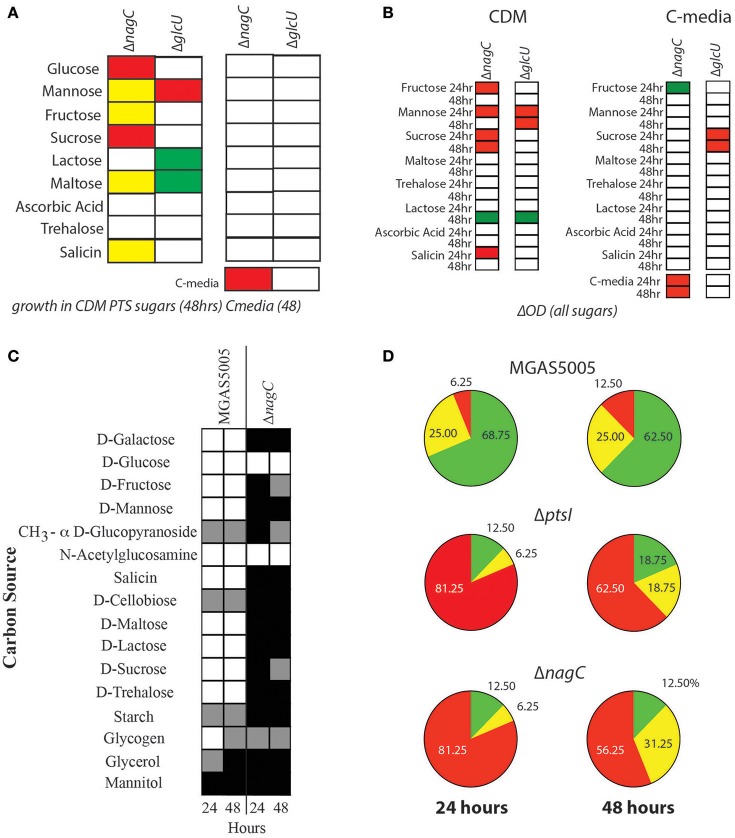
Deletion of *nagC* prohibits GAS growth in several PTS sugars. **(A)** Growth of Δ*nagC* and Δ*glcU* mutants was compared to WT MGAS5005 either in CDM + 1% PTS sugar or 0.5% glucose (left) or in C media (0.05% glucose) plus PTS sugar as described in section Materials and Methods. Growth above WT (green), 2-fold below WT (yellow), 2-fold below WT (red), and similar to WT (white) are indicated. **(B)** Growth yield (ΔOD_600_) of mutants was calculated after 24 and 48 h of growth in CDM or C-media + 1% PTS sugar. Yield similar to WT (white), lower than WT (red) or higher than WT (green) are indicated. **(C)** Carbohydrate utilization profile of the Δ*nagC* mutant was determined by the API®50CH system as described in section Materials and Methods. Positive utilization (+, white), partial utilization (±, gray), and no utilization (−, black) are shown. Readings for each strain are given for 24 h (left) and 48 h (right) are presented. **(D)** Patterns of carbohydrate utilization from **(C)** were analyzed by determining the % carbon sources listed that fall into each utilization category. Pie charts show the patterns for WT MGAS5005, Δ*ptsI*, and Δ*nagC* GAS strains at 24 and 48 h. No utilization (Red), Partial utilization (Yellow), and utilization (green) are depicted with corresponding percentages indicated.

To investigate if a non-PTS glucose metabolic pathway affects PTS function, Δ*nagC* and Δ*glcU* mutants were grown in CDM + 1% of a PTS carbohydrate as the sole carbon source for 48 h as described in section Materials and Methods. Surprisingly, the Δ*nagC* glucokinase mutant did not grow as well as wild type in glucose, fructose, sucrose and maltose (Figure 3A, Supplementary Figure 4A). The Δ*glcU* permease mutant grew slightly better than the parental MGAS5005 in both lactose and maltose, while both strains had reduced growth in mannose as compared to WT (Figure 3A, Supplementary Figure 4B). However, when these strains were grown in C media + 1% of a PTS carbohydrate or 0.5% glucose, all of these phenotypes disappeared (Figure 3A, Supplementary Figure 4). C media contains a low concentration of glucose (0.05% w/v), suggesting that Δ*nagC* and Δ*glcU* have growth defects in some PTS sugars only when they are presented as the sole carbon source. These growth defects were rescued by the presence of 0.05% (w/v) of glucose (Figure 3A) and this was reflected when comparing the total yield of these strains grown in these two different conditions (Figure 3B, Supplementary Figures 5, 6). However, since there are many differences between CDM and C media (see section Materials and Methods), it is also likely that other factors contribute to the rescue of the growth defects observed. Finally, Δ*nagC* had reduced growth and total yield in C media, further confirming that efficient glucose metabolism seems to require the presence of the glucokinase (Figures 3A,B). We next interrogated the utilization profile of the Δ*nagC* mutant to see if it reflected what was observed with growth. Using the API^®^50CH carbon utilization strips, we determined that Δ*nagC* had impaired utilization of 88% of the carbon sources tested (Figure 3C). Comparing the utilization profile of Δ*nagC* to Δ*ptsI* (Sundar et al., 2017), it is evident that both mutants have a drastic reduction in overall carbon utilization, as over 81% of carbon sources could not be utilized at 24 h, and over 56% at 48 h (Figure 3D). This suggests that similar to the PTS, NagC positively influences the metabolism of multiple carbohydrates. Importantly, utilization profiles of Δ*nagC* and Δ*glcU* rescues strains look similar to MGAS5005 (Supplementary Figure 7A).

**Figure 4:**
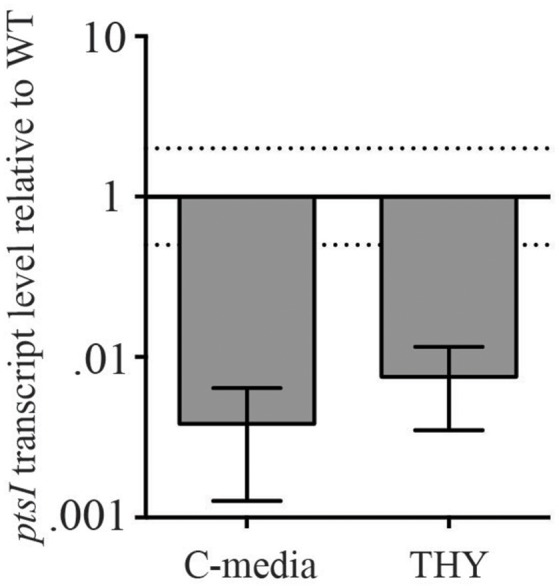
Δ*nagC* has altered expression of PTS EI (*ptsI*). Transcript level of *ptsI* in a Δ*nagC* mutant compared to MGAS5005 grown in THY and C-media, measured through qRT-PCR. Data presented are the average of three biological replicates. Dashed lines represent 2-fold expression above and below WT.

To assess whether these metabolism defects in PTS sugars were due to lower PTS expression, we assessed the transcript levels of *ptsI* in Δ*nagC* and compared it to MGAS5005. We performed qRT-PCR on RNA isolated from both strains grown in THY and C-media to late log, as indicated in section Materials and Methods. *ptsI* expression was significantly lower in Δ*nagC* than in MGAS5005 when grown in THY and C-media, suggesting that this is the possible reason for the lack of growth on PTS sugars in the glucokinase mutant (Figure 4). The mechanism by which this occurs, however, is currently unknown.

### Route of glucose uptake impacts survival of gas in human blood, but not SLS-mediated hemolysis

**Figure 5:**
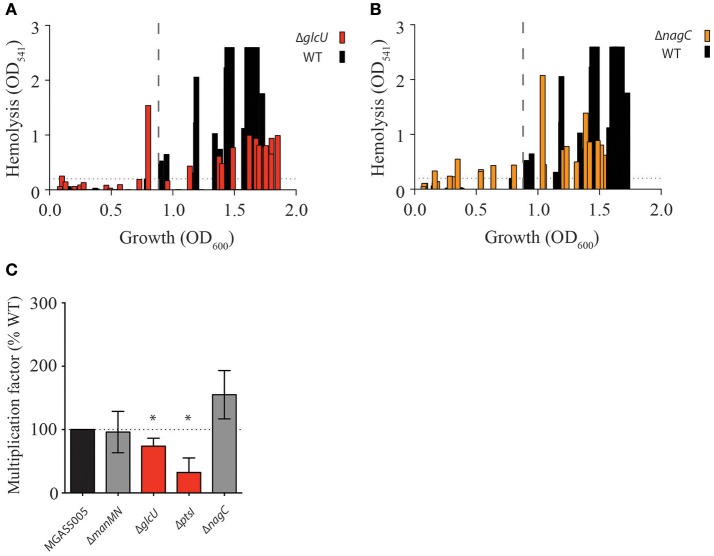
Route of glucose uptake impacts hemolysis and growth in human blood. Hemolysis assays over growth (OD_600_) in THY of WT MGAS5005 (black bars) was compared to **(A)** Δ*glcU* (red bars) and **(B)** Δ*nagC* (orange bars) as described in section Materials and Methods. Dotted lines depict the beginning of hemolytic activity in MGAS5005. **(C)** Lancefield Bactericidal Assay in whole human blood were performed and multiplication factors (MF) for each strain compared to WT MGAS5005 were calculated as described in section Materials and Methods. MF similar to WT (gray) and significantly different from WT are indicated. Data represents the average of at least three biological replicates. (^*^p-value < 0.05).

As mutants in the non-PTS glucose pathways affect glucose metabolism (Δ*glcU* and Δ*nagC*) and PTS through reducing *ptsI* expression (Δ*nagC*), we assayed whether these mutants would also display early onset of hemolysis during GAS growth. Indeed, both Δ*glcU* and, to a slightly greater extent, Δ*nagC*, exhibited hemolytic activity early in growth (Figures 5A,B). Neither mutant resembled the level of early hemolytic activity observed with Δ*ptsI*, suggesting a lesser role for these glucose utilization pathways in regulating the timely expression of SLS. However, it does show that SLS-mediated hemolysis is linked to glucose metabolism. Since both PTS and non-PTS glucose transport and metabolic pathways exhibited some degree of early hemolytic activity, we wanted to determine if this would translate to increased survival in whole human blood, as producing more SLS should allow for evasion of immune cells and access to nutrients. We found that although Δ*nagC* survives similarly to, if not better than, MGAS5005 when exposed to whole human blood, Δ*ptsI* had severely reduced survival (Figure 5C). Additionally, Δ*glcU* has a slightly reduced survival in whole human blood, suggesting that the route of glucose transport affects how GAS is able to survive in an important host environment. Although the reason for differences in Δ*nagC* and Δ*ptsI* survival in whole human blood is unknown, alterations in gene expression between the two strains as a result of forcing glucose to be metabolized by certain pathway could be one reason.

### A Δ*nagC* mutant exhibits increased lesion severity compared to WT MGAS5005

**Figure 6:**
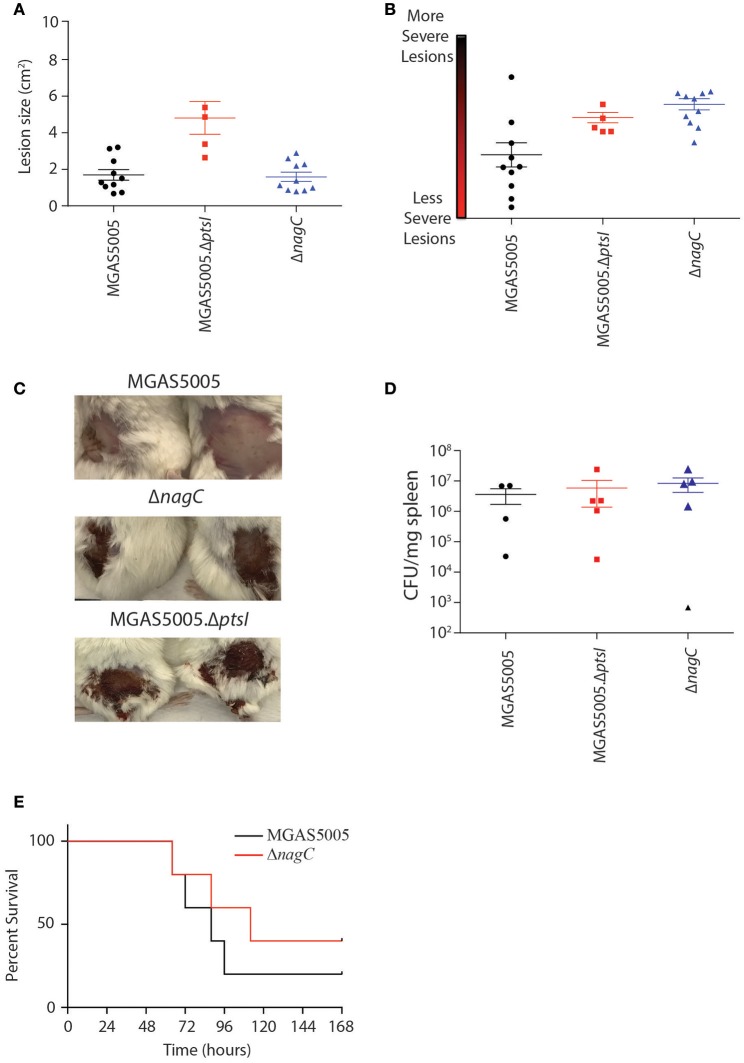
Contribution of PTS and non-PTS glucose utilization pathways in a murine model of GAS soft tissue infection. Female 5 to 7-week old CD-1 mice were injected subcutaneously with approximately 1 × 10^9^ CFU of either MGAS5005 (black circles), Δ*ptsI* (red squares), or Δ*nagC* (blue triangles) were assessed for lesion size **(A)** and severity **(B)** at 48 h post infection (hpi) as described in section Materials and Methods. Each data point represents a separate animal. **(C)** Representative images of 2 lesions from each strain taken at 48 hpi. **(D)** Dissemination was assessed by enumeration of bacterial load in spleens at 48 h and represented as CFU/mg spleen. Each data point represents a separate animal. **(E)** Mice infected with WT MGAS5005 (black) and Δ*nagC* (red) were monitored for survival over 7 days (*n* = 10, 5 for Δ*ptsI*). Data was analyzed for significance using the log-rank.

To determine if the route of glucose metabolism affects overall GAS pathogenesis, we injected approximately 1 × 10^8^ CFU of MGAS5005, Δ*nagC*, and Δ*ptsI* subcutaneously in CD-1 mice and measured lesion size, severity, and dissemination (CFU/mg spleen) for all three strains as described in section Materials and Methods. As observed previously (Gera et al., 2014), Δ*ptsI* exhibited larger lesions as compared to parental MGAS5005, while Δ*nagC*-infected animals showed lesions comparable in size to WT at 48 hpi (Figure 6A). However, the Δ*nagC*-induced lesions were more severe than those of WT MGAS5005, and comparable to Δ*ptsI*-infected mice (Figures 6B,C). These results correlate with the hemolytic profile of all three strains, where Δ*ptsI* shows early and robust hemolysis, while Δ*nagC* hemolysis was early, but not as robust as Δ*ptsI* (Figure 5B). All three strains showed similar CFU/mg of spleen at 48 h, suggesting that dissemination from the local site of infection was comparable (Figure 6D). This was somewhat unexpected as Δ*ptsI* did not survive in whole human blood and may indicate a human-specific phenotype. It also suggests that the PTS and *nagC* help control hemolytic activity at the local site of skin infection, but this does not translate to greater dissemination, resulting in similar lethality of infected mice between the two mutant strains and MGAS5005 (Figure 6E) (Gera et al., 2014). In conclusion, glucose metabolism through the PTS is important for survival in whole human blood whereas impaired glucose utilization in general leads to the early production of SLS, resulting in more severe lesions *in vivo*.

## Discussion

In this study, we generated mutants in predicted glucose utilization pathways and compared the contribution of non-PTS pathways through glucokinase (*nagC*) to the PTS pathway for overall glucose metabolism. We found that elimination of the non-PTS glucose metabolic pathway led to a more drastic reduction in growth on glucose as the sole carbon source as compared to effects of eliminating the PTS alone. Interestingly, we discovered that deletion of the glucokinase *nagC* also leads to decreased growth in PTS sugars, likely resulting from the decreased expression of *ptsI* in this background. Altering metabolic pathways for glucose transport affected the temporal activity of Steptolysin S (SLS), as both Δ*nagC* and Δ*glcU* showed an early hemolytic profile during growth. Although the route of glucose transport did not affect the onset of early hemolysis, it did affect survival in human blood as the Δ*ptsI* mutant is unable to survive whereas the Δ*nagC* mutant did. Early onset of hemolysis with Δ*nagC* translated to an increase in lesion severity, but not in lesion size, at the site of infection during soft tissue infection of mice. In addition, dissemination to the spleen and systemic lethality was not affected by the absence of NagC glucokinase. Thus, we have established that there are at least two transport systems for glucose that are interconnected and influence the expression of the toxin SLS.

Different types of sugar transporters, both PTS and non-PTS, have been shown to transport glucose in bacteria (Castro et al., 2009; Vitko et al., 2016). In *S. aureus*, a double mutant in the glucokinase *nagC* (non-PTS transporters) and *ptsI* (PTS transporters) ablated glucose metabolism (Vitko et al., 2016) indicating that they represent the sole pathways for glucose metabolism. In GAS, a Δ*nagC* mutant showed a greater defect after growth for 48 h in all concentrations of glucose when compared to a Δ*ptsI* (Figure 2), indicating that a non-PTS pathway is more important in overall glucose metabolism under the tested sugar concentrations. Although GAS possesses a homolog of the non-PTS glucose transporter GlcU (*glcU*) important for glucose metabolism in *S. aureus* (Vitko et al., 2016), inactivation of *glcU* in GAS leads to only a modest growth defect in all glucose concentrations tested (Figure 2). Coupled with the fact that a Δ*glcU*Δ*ptsI* double mutant was still able to grow on glucose (Supplementary Figure 7B), this strongly suggests the presence of an additional glucose transporter(s) that have yet to be identified. A Δ*nagC*Δ*ptsI* double mutant would allow us to confirm this; however, we have been unable to find a carbon source that would facilitate growth of a GAS Δ*ptsI*Δ*nagC* double mutant. Since *S. aureus* has a much more robust metabolic capacity compared to GAS, this may explain why substrates allowing growth of the GAS Δ*nagC*Δ*ptsI* double mutant were more readily identifiable.

Unexpectedly, a GAS Δ*nagC* mutant displayed trouble growing on the PTS sugars mannose, fructose, sucrose, and maltose, possibly due to a reduction in *ptsI* expression (Figures 3, 4). Although the mechanism of this transcriptional repression is unknown, it is possible that the Δ*nagC* mutant results in a reduction in intracellular PEP pools that then leads to lowered PTS activity. Since growth defects of the Δ*nagC* mutant in PTS sugars disappeared when using C media (low glucose-high peptide) in place of CDM (no carbon source), it is possible that growing Δ*nagC* in low glucose (0.5%) allows for the generation of enough PEP to stimulate PTS expression and allow for growth on PTS sugars.

Invasive GAS (iGAS) infections involve the bloodstream to disseminate from the local site of infection to normally sterile sites in the body, leading to life-threatening systemic infection. Since human blood is glucose rich, we interrogated whether the route of glucose transport affected survival of GAS in whole human blood. Surprisingly, the Δ*nagC* glucokinase mutant impacting non-PTS glucose transport was able to survive comparable to wild type MGAS5005 whereas the Δ*ptsI* PTS mutant was not (Figure 5). These data strongly suggest that PTS-mediated glucose transport is important for GAS survival during bacteremia. PTS transporters have been shown to be important for virulence in many pathogens, even in GAS, so it is possible that these either transport glucose as well, or somehow influence PTS-mediated glucose transport (Lun and Willson, 2004; Abranches et al., 2006; Stevens et al., 2010; Aké et al., 2011; McAllister et al., 2012; Wu et al., 2012). One important caveat is that the Δ*ptsI* mutant not only affects glucose transport, but also impacts the metabolism of 10 non-PTS carbon sources (Gera et al., 2014). Therefore, it is possible that the GAS Δ*ptsI* mutant has broader perturbations outside of glucose transport that are required for survival in blood. Evidence for this lies in the fact that blood contains detectable levels of 8 PTS sugars (Wishart et al., 2007, 2009, 2013). However, the concentrations these sugars are present in blood (and average ~1–64 μM) are often 100-fold below the concentration of glucose (average of ~4,800 μM) (Wishart et al., 2007, 2009, 2013). Even so, it is certainly still possible these low concentrations are enough to influence GAS survival.

A number of toxins produced by bacterial pathogens have increased expression under low nutrient conditions (Dineen et al., 2007; Seidl et al., 2008; Antunes et al., 2011). In GAS MGAS5005, we observed that secreted SLS activity in cell-free supernatants is observed under lower sugar environments (Figure 1). Given that the *sag* operon appears in the published core CcpA regulon (DebRoy et al., 2016), we hypothesized that disruptions in glucose transport might lead to early SLS-mediated hemolysis. Supporting this concept, both Δ*nagC* and Δ*glcU* exhibit early hemolytic activity, although not to the same magnitude as was seen with Δ*ptsI* (Figure 5). Disruption of glucokinase (Δ*nagC*) also led to an increase in lesion severity in the subcutaneous murine model of soft tissue infection as compared to wild type MGAS5005. This suggests not only that lesion severity correlates with production of SLS, as was shown previously (Gera et al., 2014), but that the regulation of SLS activity is affected by NagC by an undefined mechanism. We speculate that production of SLS may be a response to a low nutrient environment, perhaps in an effort to obtain more nutrients through the destruction of host cells.

Dissemination of wild type MGAS5005, Δ*ptsI*, and Δ*nagC* during murine soft-tissue infection was quite similar based on CFU/mg spleen after 2 days (Figure 6B). This was unexpected for Δ*ptsI*, since it does not survive as well as MGAS5005 or Δ*nagC* during growth in whole human blood (Figure 5C). Therefore, the requirement of the PTS in whole blood survival is potentially human-specific. This may not be too surprising given that we showed mutants in the PTS fructose metabolism operon (Δ*fruBAC*) were able to grow in mouse blood but not human blood (Valdes et al., 2016). Survival, lesion size, and dissemination of mice infected with MGAS5005 and the Δ*nagC* mutant were comparable, suggesting that the role of glucokinase in GAS pathogenesis may be localized to the site of infection.

Overall, this suggests that the relevance of the route of glucose metabolism to GAS pathogenesis varies with the host and specific niche. Forcing glucose to be transported through one route over the other appears to impact the ability of GAS to survive *in vivo*, either due to the efficiency of glucose transport or the expression of other important pathogenic networks. A likely scenario is that restricting glucose uptake through the PTS changes glucose from a preferred sugar to a PTS-inducing sugar that can affect the phosphorylation of PTS-regulatory domains (PRD) on PRD-containing virulence regulators (PCVR)s, thereby changing their activity in regulating gene expression. This would not be the case for the Δ*ptsI* mutant, where PTS phosphorylation of PRDs cannot occur. GAS may use the affinity of both systems for glucose transport, coupled with the ability to phosphorylate PCVRs in response to inducing sugars, to regulate virulence programs in different environments.

**Figure 7:**
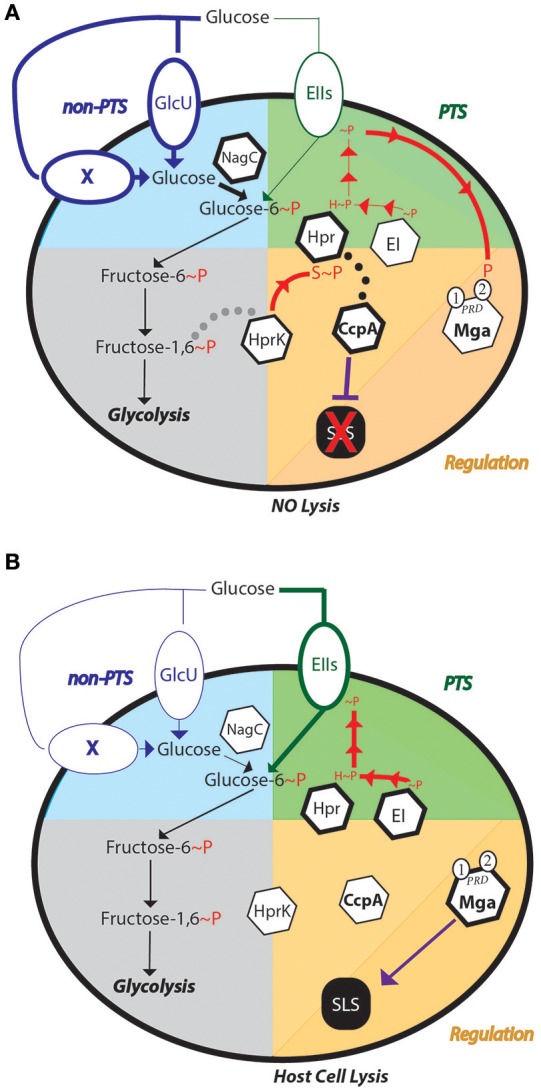
Regulation of SLS-mediated hemolysis of GAS in different glucose environments. Models are presented for the impact of both **(A)** high and **(B)** low glucose on the routes of uptake, CcpA-mediated carbon catabolite repression and SLS-mediated hemolysis. The non-PTS (blue) and PTS (green) glucose metabolic pathways are represented as the upper quadrants, while Glycolysis (gray) is in the lower left and regulation of SLS (orange) in the lower right. Dotted lines represent potential protein interactions and solid lines depict either metabolic reactions or regulation. P represents phosphorylation, with H~P indicating histidine phosphorylation and S~P serine that phosphorylation. X signifies a predicted but unidentified non-PTS transporter for glucose. Mga is a PRD-containing virulence regulator that interacts with the PTS and can regulate SLS expression under low glucose conditions. Hemolytic outcomes are indicated at the bottom.

Based on the evidence presented here and in the literature, we propose the following hypothetical model for SLS-mediated hemolysis regulation in response to glucose levels. When a high amount of glucose is present in the environment (Figure 7A), we hypothesize that glucose is transported primarily through the non-PTS glucose transporters (Blue section), as these enzymes are active in early exponential phase of growth (unpublished). Transport may still occur through the PTS, however, this is unlikely as *ptsI* is actively expressed during late log-phase of growth (unpublished). The rapid uptake of glucose would lead to a buildup of fructose-1,6-bisphosphate, a known trigger for HprK to phosphorylate HPr on its Serine-46 residue (Deutscher, 2008). HPr-Ser~P could then be a cofactor for CcpA to repress SLS-mediated hemolysis by preventing expression of the *sag* operon (DebRoy et al., 2016). The exact mechanism for this is currently unknown, in that even though the *sag* operon is included in the CcpA regulon and a Δ*ccpA* mutant exhibits early hemolysis (Kinkel and McIver, 2008; DebRoy et al., 2016), CcpA does not bind to the *sag* promoter *in vivo* (Kietzman and Caparon, 2010). Therefore, CcpA-mediated repression of the *sag* operon is likely indirect. Additionally, since glucose would primarily be transported by the non-PTS metabolic pathway(s), EII subunits would likely remain phosphorylated, which could present the opportunity to phosphorylate the PCVR Mga, a global transcriptional regulator known to regulate many virulence factors, including *emm* (Hondorp et al., 2013). However, when glucose levels become low, the PTS becomes the primary mode of glucose transport, as suggested by the mannose-specific EII shown to be a high affinity glucose transporter in *S. pneumoniae* (Fleming and Camilli, 2016). Expression of many PTS EII components occur in late-log phase, which correlates with low glucose levels (unpublished). As glucose levels lower and PTS activity ramps up (Figure 7B), HPr-Ser~P can either be phosphorylated by EI, or the phosphate on the serine can be cleaved by HprK. This leads to derepression of the *sag* operon, as CcpA is no longer interacting with HPr. As the phosphorelay continues to shuttle phosphates to the EIIBs that phosphorylate the incoming sugar, Mga can no longer be phosphorylated, leading to further activation of the *sag* operon. This is thought to occur as Mga was found to activate *sag* gene expression in low glucose conditions (Valdes and McIver, in press), although direct regulation has not been shown. This would lead to robust SLS-mediated hemolysis in low-glucose conditions (Figure 1).

## Conclusions

This study shows that the route of glucose transport affects the pathogenesis of GAS in different host tissues, potentially due to differences in growth or toxin production profiles when glucose is transported through one pathway over the other.

## Author contributions

GS, YLB, and KM: conceived and designed the research plan, supervised the project, analyzed the data and interpreted the results; GS: performed most of the experiments with technical contributions from EI, RB, and AS; GS, YLB, and KM: wrote the manuscript. All authors read and approved the final manuscript.

### Conflict of interest statement

The authors declare that the research was conducted in the absence of any commercial or financial relationships that could be construed as a potential conflict of interest.
