# Supplementary material for: Route of Glucose Uptake in the Group a Streptococcus Impacts SLS-Mediated Hemolysis and Survival in Human Blood
Source: Front Cell Infect Microbiol. 2018 Mar 14;8:71. doi: 10.3389/fcimb.2018.00071 (PMC5861209; doi:10.3389/fcimb.2018.00071)
Supplement: Supplementary file 1 [file Table1.PDF]

Supplemental Table 1. Bacterial strains

Bacterial Strains	***Relevant Description***	***Reference***
E. coli		
DH5α	*hsdR17 recA1 gyrA endA1 relA1*	(Hanahan & Meselson, 1983)
S. pyogenes **(GAS)**		
MGAS5005	M1T1 *covS*	(Sumby *et al.*, 2005)
MGAS5005.Δ*ptsI*	Δ*ptsI* mutant in MGAS5005	(Gera *et al.*, 2014)
MGAS5005.Δ*manMN*	MGAS5005 i.i. of *manMN*	(Sundar, 2017)
MGAS5005.Δ $manMN_R$	MGAS5005.Δ*manMN* rescue	(Sundar, 2017)
MGAS5005.Δ*nagC*	MGAS5005 i.i. of *nagC*	This study
MGAS5005.Δ*glcU*	MGAS5005 i.i. of *glcU*	This study
MGAS5005.Δ*glcU*Δ*ptsI*	MGAS5005 i.i. of *glcU*/a.e. of *ptsI*	This study
MGAS5005.Δ $nagC_R$	MGAS5005.Δ*nagC* revertant	This study
MGAS5005.Δ $glcU_R$	MGAS5005.Δ*glcU* revertant	This study

* i.i = Insertional Inactivation
** a.e.= Allelic Exchange

Supplemental Table 2. Plasmids

Plasmids	*Relevant Description*	*Reference*
pCRS	Temperature-sensitive conditional vector; Sp^R	(Le Breton & McIver, 2013)
pCRK	Temperature-sensitive conditional vector; Km^R	(Le Breton & McIver, 2013)
pKSM968	nagC mutagenic plasmid with nonpolar Km^R	This study
pKSM969	pCRS glcU mutagenic plasmid for i.i.; pCRS	This study
pKSM970	glcU mutagenic plasmid for i.i.; pCRK	This study

Supplemental Table 3. Primers

Primer	***Sequence 5' to 3'***	***Target***	***Reference***
oAX1311-1	CCC**GGATCC**AGAAATCCCTTTGACTGAA	*nagC*	This study
oAX1311-2k	GGTGATATTCTCATTTTAGCCATGAGGTG<br>TTCTCCTTTATTATAG	*nagC*	This study
oAX1311-3k	ATTTTACTGGATGAATTGTTTTAGCCATG<br>GACCGCTAAATCCCTC	*nagC*	This study
oAX1311-4	CCC**GGATCC**TTGATGGCTGGTCTTGGCTC	*nagC*	This study
Spy1856 F	CCC**GGATCC**GATGACACTGCAACTTTGG	*glcU*	This study
Spy1856 R	CCC**GGATCC**CCAATGCCCCAAAGTAATC	*glcU*	This study
oAX1311-V	CCATTACGACAATATTCCTTGG	5' of *nagC*	This study
Spy1856 mutantconf1	CCATTACGACAATATTCCTTGG	5' of *glcU*	This study
ptsI M1 RT L	CGGAAACCAAGGAATGGAT	*ptsI*	(Gera et al., 2014)
ptsI M1 RT R	TGGCAAACCTGTTGTGGTT	*ptsI*	(Gera et al., 2014)
gyrA M1 RT-L	CGACTTGTCTGAACGCCAAAGTC	*gyrA*	(Ribardo & McIver, 2006)
gyrA M1 RT-R	ATCACGTTCCAAACCAGTCAAAC	*gyrA*	(Ribardo & McIver, 2006)
1201	AACAGCTATGACCATGATTACG	*M13*	Genewiz
1211	GTTGTAAAACGACGGCCAGT	*M13*	Genewiz

* bp in bold denote restriction sites

** Underlined bp denote areas of overlap with a resistance cassette for PCR soeing

Supplemental References

Gera, K., T. Le, R. Jamin, Z. Eichenbaum & K. S. McIver, (2014) The Phosphoenolpyruvate Phosphotransferase System in Group A Streptococcus Acts To Reduce Streptolysin S Activity and Lesion Severity during Soft Tissue Infection. *Infect Immun* **82**: 1192-1204.

Hanahan, D. & M. Meselson, (1983) Plasmid screening at high colony density. *Methods Enzymol* **100**: 333-342.

Le Breton, Y. & K. S. McIver, (2013) Genetic Manipulation of *Streptococcus pyogenes* (The Group A Streptococcus, GAS). *Curr Prot Microbiol* **In Press**.

Ribardo, D. A. & K. S. McIver, (2006) Defining the Mga regulon: comparative transcriptome analysis reveals both direct and indirect regulation by Mga in the group A streptococcus. *Mol Microbiol* **62**: 491-508.

Sumby, P., S. F. Porcella, A. G. Madrigal, K. D. Barbian, K. Virtaneva, S. M. Ricklefs, D. E. Sturdevant, M. R. Graham, J. Vuopio-Varkila, N. P. Hoe & J. M. Musser, (2005) Evolutionary origin and emergence of a highly successful clone of serotype M1 group A streptococcus involved multiple horizontal gene transfer events. *J Infect Dis* **192**: 771-782.

Sundar, G., Islam, E., Gera K., Le Breton, Y., McIver KS. , (2017) A PTS EII mutant library in Group A Streptococcus identifies a promiscuous man-family PTS transporter influencing SLS-mediated hemolysis. *Mol Microbiol* **103**: 518-533.
