# Supplementary material for: Route of Glucose Uptake in the Group a Streptococcus Impacts SLS-Mediated Hemolysis and Survival in Human Blood
Source: Front Cell Infect Microbiol. 2018 Mar 14;8:71. doi: 10.3389/fcimb.2018.00071 (PMC5861209; doi:10.3389/fcimb.2018.00071)
Supplement: Supplementary file 2 [file Image1.PDF]

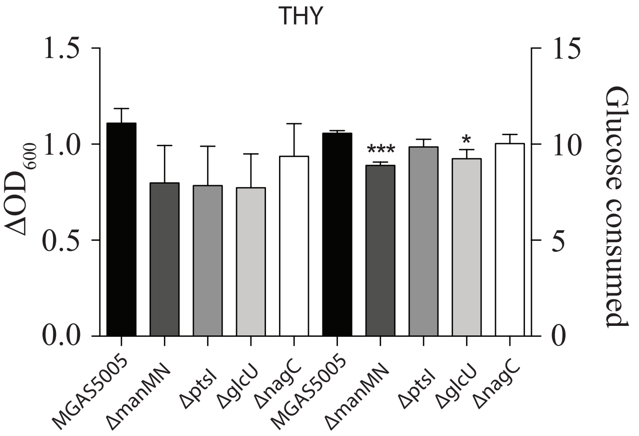

Supplemental Figure 1: Glucose consumed by GAS strains during growth. Total yield was measured as indicated in Methods. Data is represented as the average of at least three replicates. Total amount of sugar consumed was calculated by measuring the concentration of sugar in the medium both before and after growth.

A. Δ*manM*

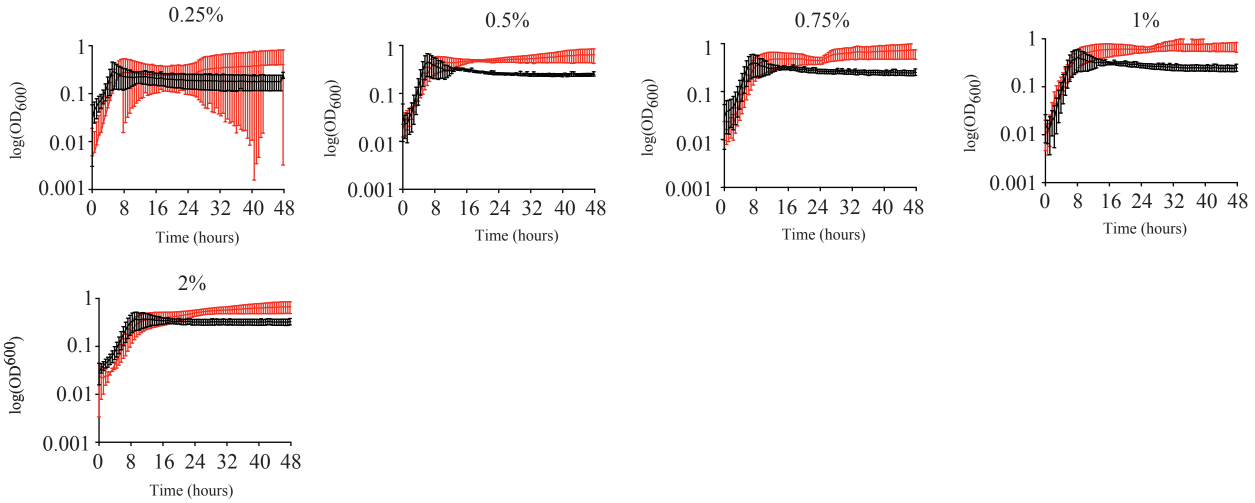

B. Δ*glcU*

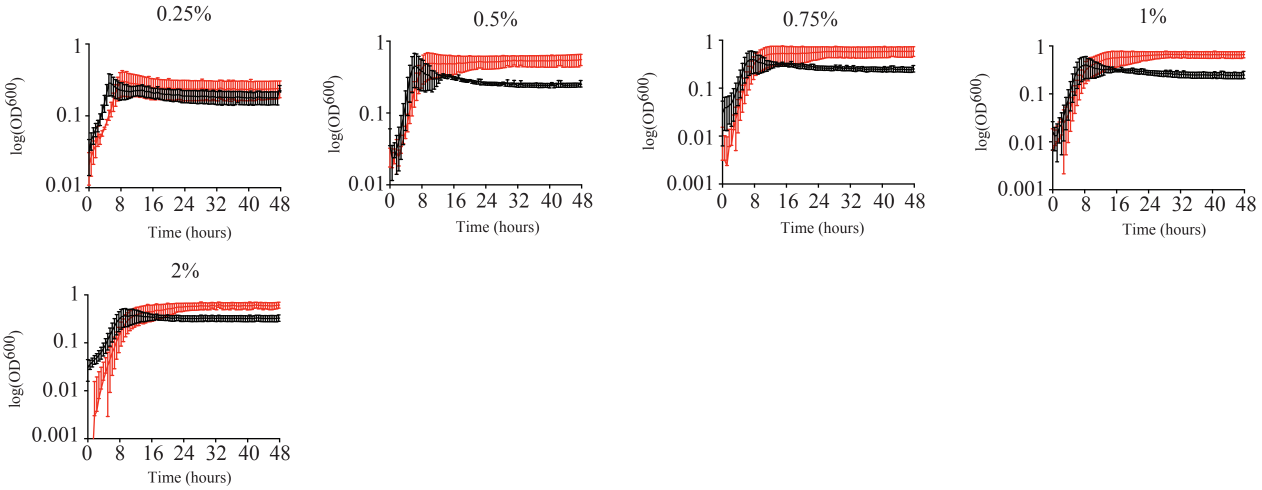

C. Δ*ptsI*

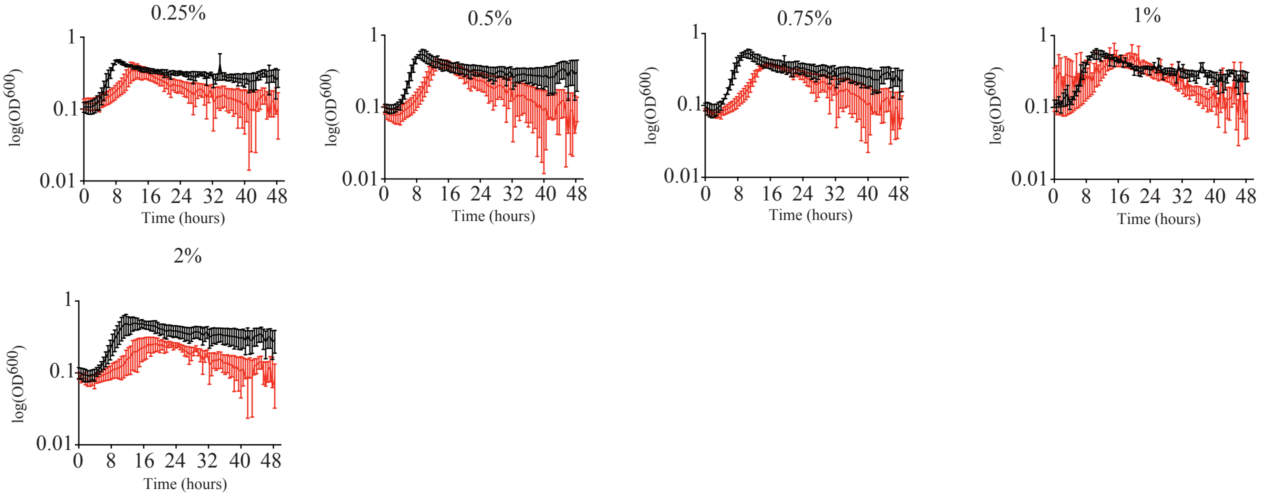

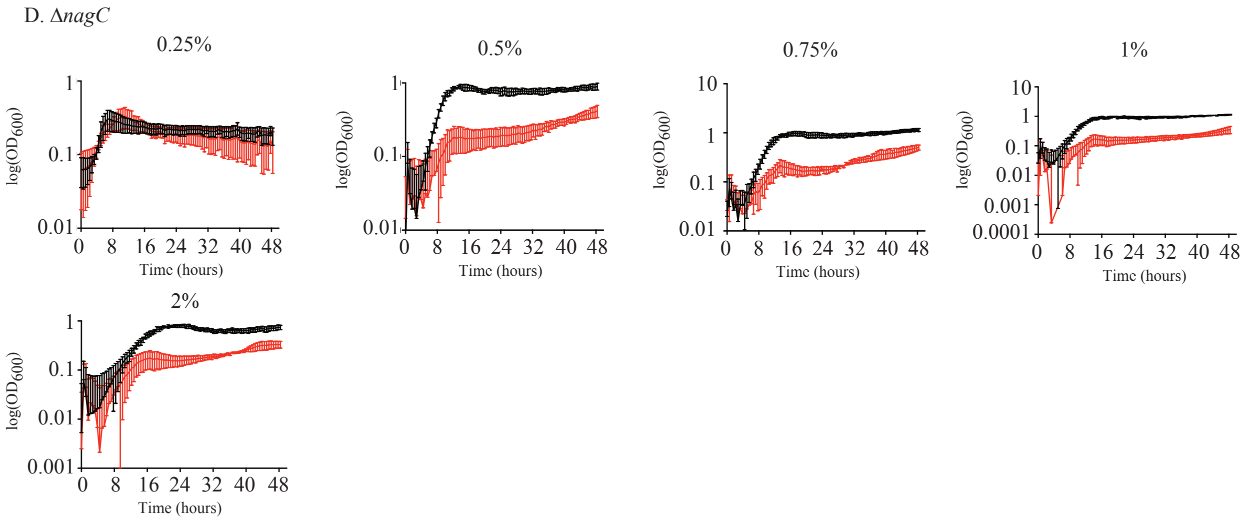

Supplemental Figure 2: Growth of GAS strains in different concentrations of glucose. GAS strains were grown in CDM plus glucose as indicated in Methods. MGAS5005 growth is depicted in black, and mutant strain growth is indicated in red. Error bars illustrate the standard error of the mean. Data represents the average of at least three biological replicates. Percentage of glucose added to CDM is indicated above each graph.

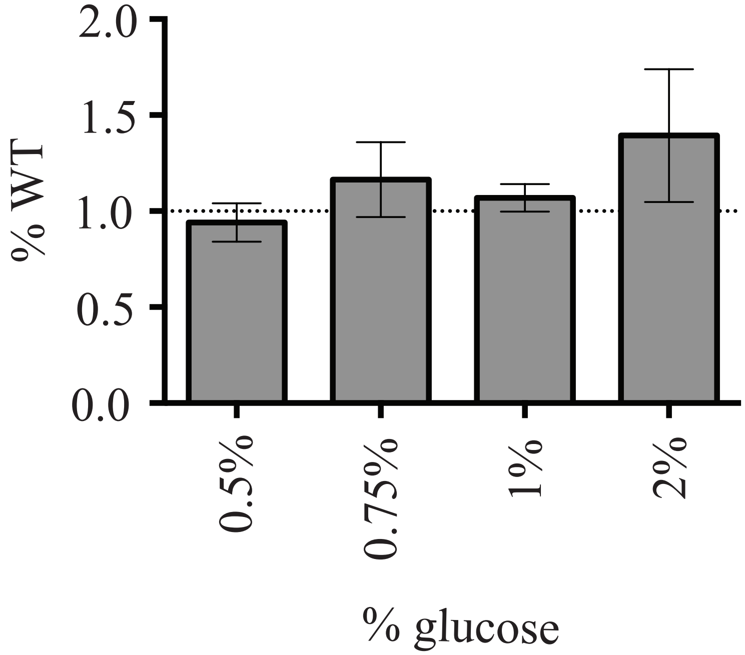

Supplemental Figure 3. **Δ*nagC*$_R$ grows similarly in CDM + various % glucose to MGAS5005.** Total yield depicted was calculated as indicated in Methods, and the data shown is the average of at least three biological replicates.

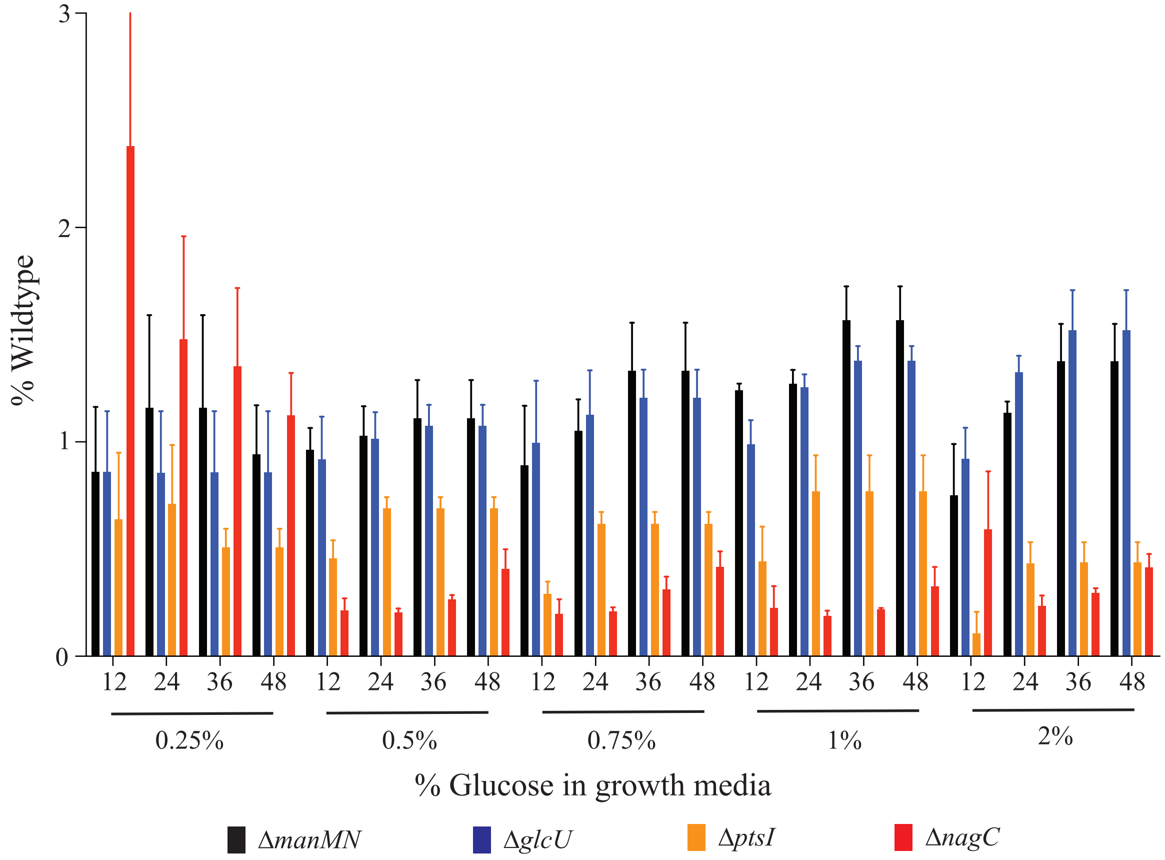

Supplemental Figure 4: Total yield of GAS strains grown in various concentrations of glucose. GAS strains are represented by the colors depicted. Comparisons graphed between MGAS5005 and the mutant strains represent analysis at 12, 24, 36, and 48 hours. Data is the average of at least three replicates.

A. Δ*nagC*

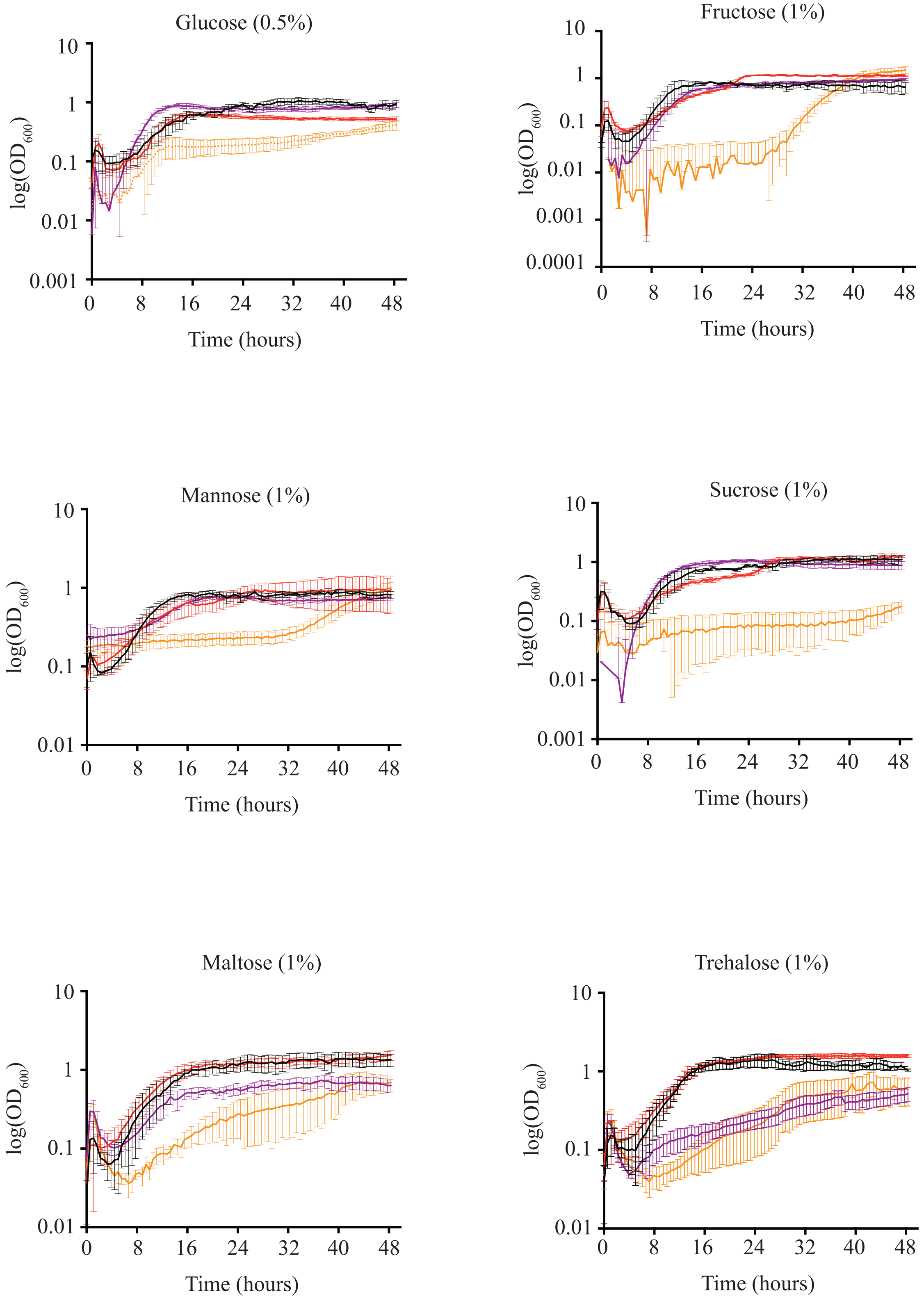
Glucose (0.5%)
Fructose (1%)
Mannose (1%)
Sucrose (1%)
Maltose (1%)
Trehalose (1%)
log(OD600)
Time (hours)
10
1
0.1
0.01
0.001
0.0001
0
8
16
24
32
40
48

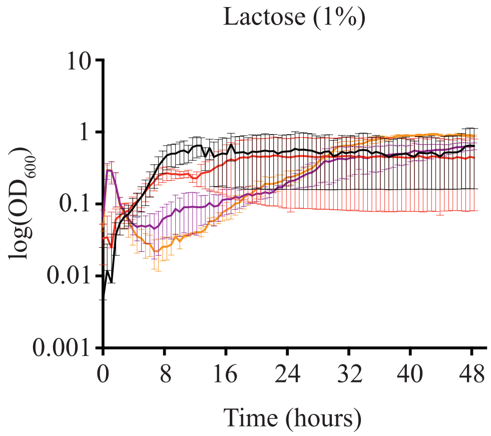
Lactose (1%)
10
1
0.1
0.01
0.001
log(OD600)
0
8
16
24
32
40
48
Time (hours)

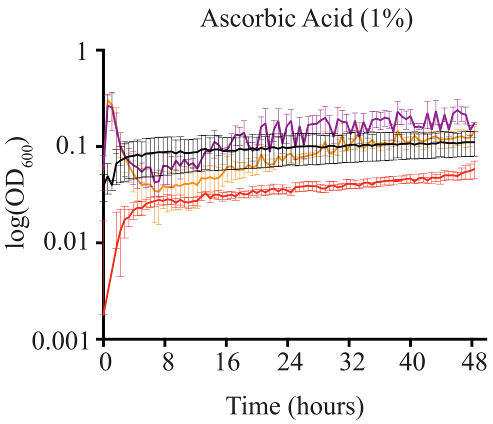
Ascorbic Acid (1%)
1
0.1
0.01
0.001
log(OD600)
0
8
16
24
32
40
48
Time (hours)

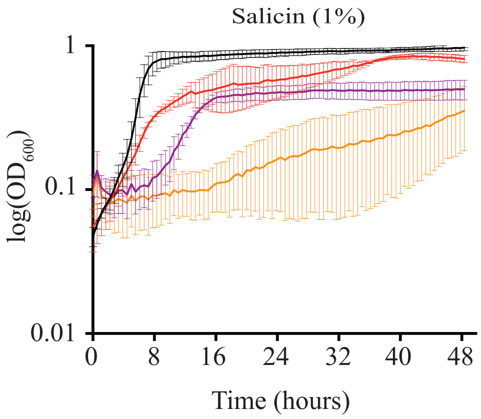
Salicin (1%)
1
0.1
0.01
log(OD600)
0
8
16
24
32
40
48
Time (hours)

B. Δ*glcU*

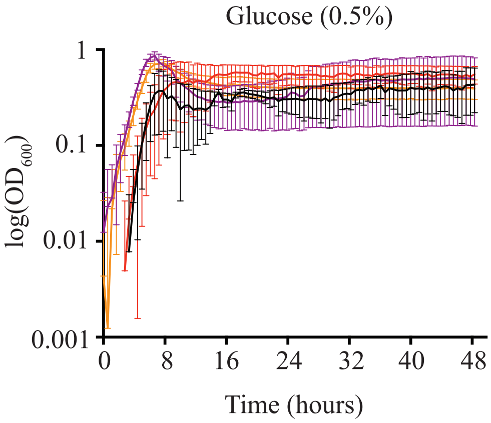
Glucose (0.5%)
1
0.1
0.01
0.001
log(OD600)
0
8
16
24
32
40
48
Time (hours)

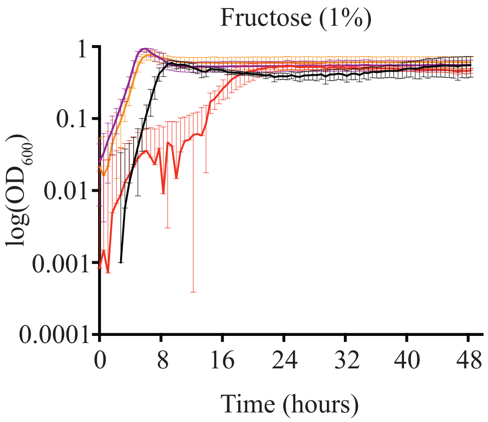
Fructose (1%)
1
0.1
0.01
0.001
0.0001
log(OD600)
0
8
16
24
32
40
48
Time (hours)

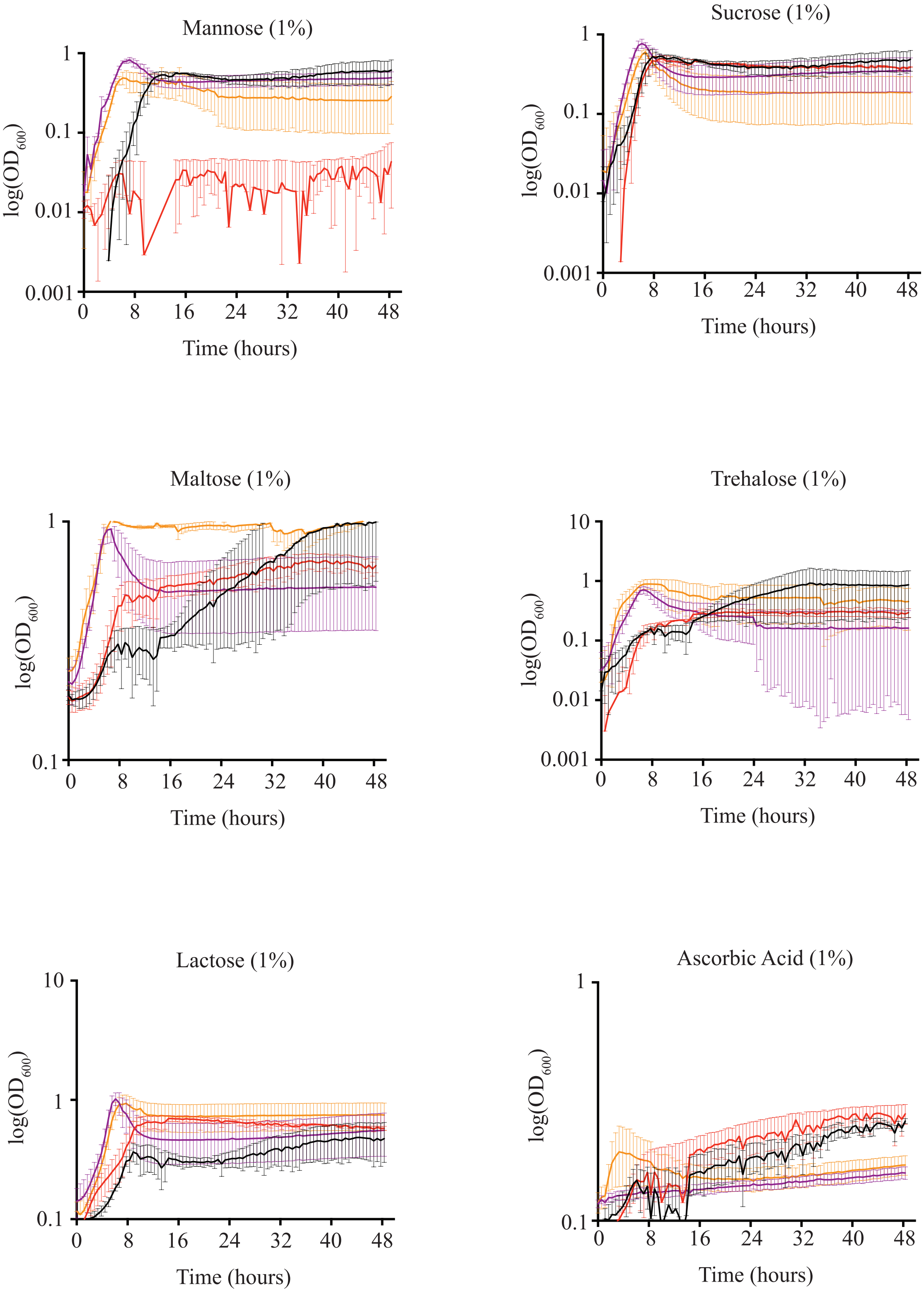
Mannose (1%)
Sucrose (1%)
Maltose (1%)
Trehalose (1%)
Lactose (1%)
Ascorbic Acid (1%)
log(OD600)
Time (hours)
0
8
16
24
32
40
48
10
1
0.1
0.01
0.001

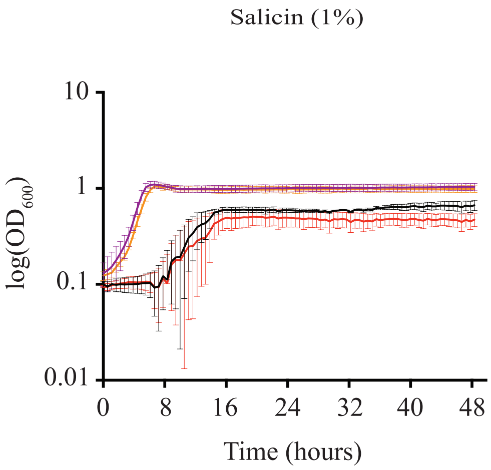

Supplemental Figure 5: Δ*nagC* and Δ*glcU* grown in 1% PTS carbohydrates. GAS strains were grown in CDM or C-media plus PTS sugars as indicated in Methods. MGAS5005 growth is depicted in black (C-media) and purple (CDM). Mutant growth is illustrated in red (C-media) and orange (CDM). Data represents the average of at least three biological replicates.

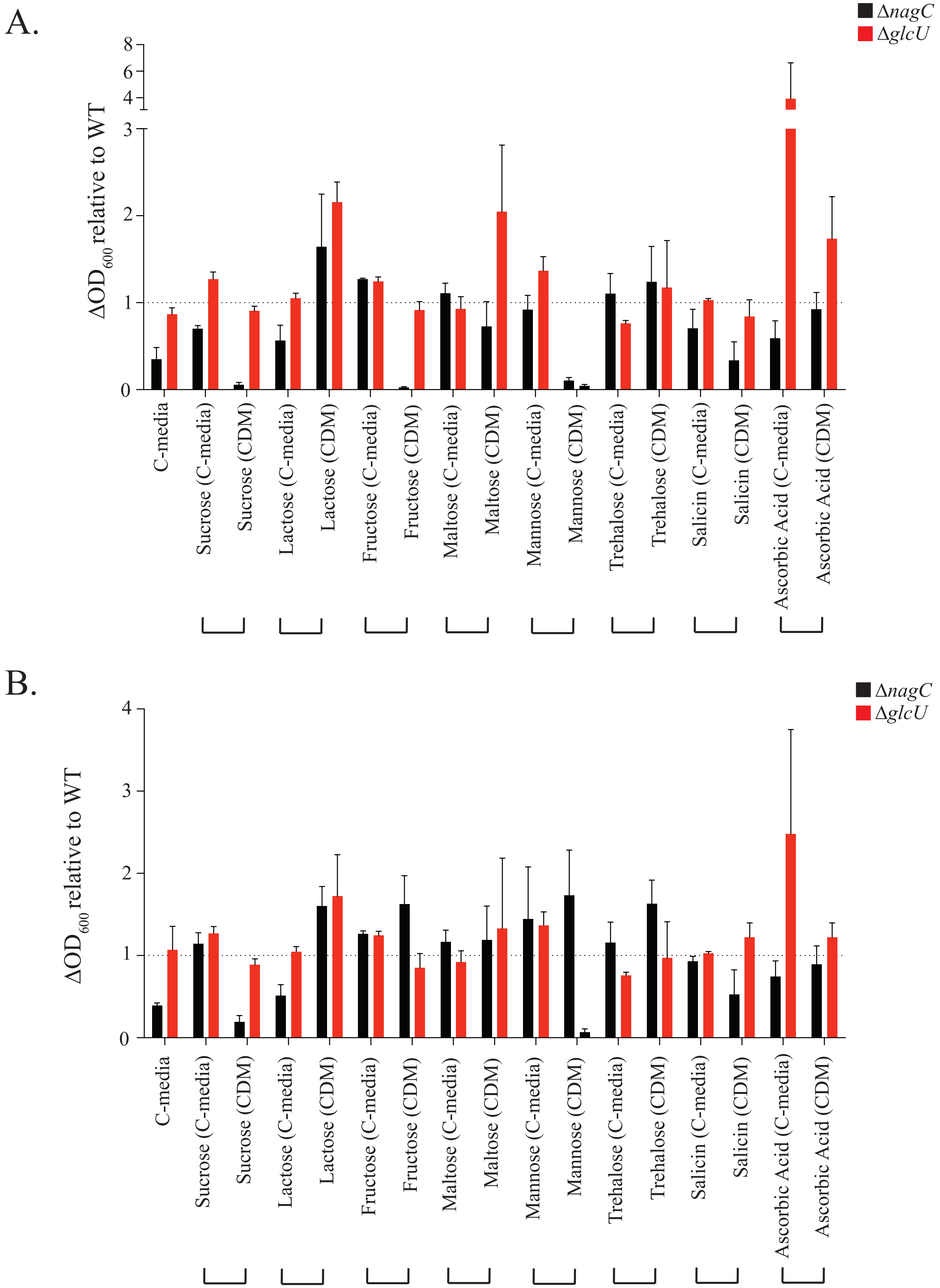

A.
ΔnagC
ΔglcU
ΔOD600 relative to WT
0
1
2
3
4
6
8
C-media
Sucrose (C-media)
Sucrose (CDM)
Lactose (C-media)
Lactose (CDM)
Fructose (C-media)
Fructose (CDM)
Maltose (C-media)
Maltose (CDM)
Mannose (C-media)
Mannose (CDM)
Trehalose (C-media)
Trehalose (CDM)
Salicin (C-media)
Salicin (CDM)
Ascorbic Acid (C-media)
Ascorbic Acid (CDM)
B.
ΔnagC
ΔglcU
ΔOD600 relative to WT
0
1
2
3
4
C-media
Sucrose (C-media)
Sucrose (CDM)
Lactose (C-media)
Lactose (CDM)
Fructose (C-media)
Fructose (CDM)
Maltose (C-media)
Maltose (CDM)
Mannose (C-media)
Mannose (CDM)
Trehalose (C-media)
Trehalose (CDM)
Salicin (C-media)
Salicin (CDM)
Ascorbic Acid (C-media)
Ascorbic Acid (CDM)

Supplemental Figure 6: Total yield of Δ*nagC* and Δ*glcU* grown in PTS carbohydrates. GAS strains were grown in PTS sugars with either CDM or C-media as the base media as indicated in Methods. Data are represented as the average of at least biological replicates, shown relative to MGAS5005. An ΔOD_{600} closer to 1 indicates a total yield similar to MGAS5005. Total yields for Δ*nagC* is shown in black, and Δ*glcU* is shown in red. Each bracket groups the same PTS sugar in two different medias. Total yields for both A) 24 and B) 48 hours are illustrated.

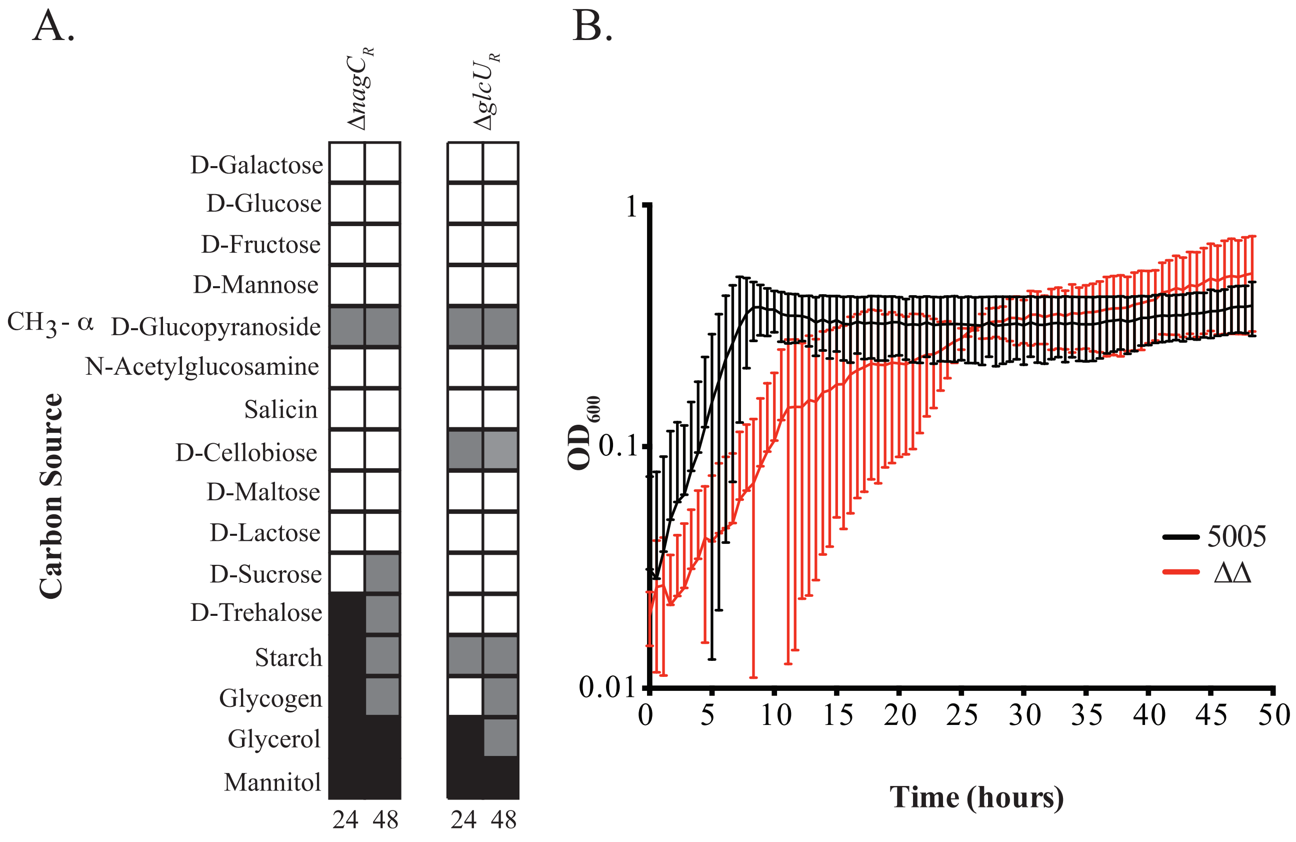

Supplemental Figure 7. A) Carbohydrate utilization profile of the Δ$nagC_R$ and Δ$glcU_R$ was determined by the API®50CH system as described in Methods. Positive utilization (+, white), partial utilization (+/-, grey), and no utilization (-, black) are shown. Readings are given for 24 h (left) and 48 h (right) are presented. B) Growth of ΔglcUΔptsI (ΔΔ) in 0.5% glucose over 48 hours. Error bars indicate one standard deviation.
